# Selenium Status in Paediatric Patients with Neurodevelopmental Diseases

**DOI:** 10.3390/nu14122375

**Published:** 2022-06-08

**Authors:** Christian L. Görlich, Qian Sun, Viola Roggenkamp, Julian Hackler, Sebastian Mehl, Waldemar B. Minich, Angela M. Kaindl, Lutz Schomburg

**Affiliations:** 1 Institute for Experimental Endocrinology, Charité-Universitätsmedizin Berlin, 13353 Berlin, Germany; christian.goerlich@charite.de (C.L.G.); qian.sun@charite.de (Q.S.); julian.hackler@charite.de (J.H.); sebastian.mehl@charite.de (S.M.); waldemar.minich@charite.de (W.B.M.); 2 Center for Chronically Sick Children (SPZ), Charité-Universitätsmedizin Berlin, 13353 Berlin, Germany; viola.roggenkamp@web.de (V.R.); angela.kaindl@charite.de (A.M.K.); 3 Department of Pediatric Neurology, Charité-Universitätsmedizin Berlin, 13353 Berlin, Germany; 4 Institute of Cell Biology and Neurobiology, Charité-Universitätsmedizin Berlin, 13353 Berlin, Germany

**Keywords:** trace element, inflammation, biomarker, micronutrient, neurodevelopment

## Abstract

Neurodevelopmental diseases are often associated with other comorbidities, especially inflammatory processes. The disease may affect the trace element (TE) status, which in turn may affect disease severity and progression. Selenium (Se) is an essential TE required for the biosynthesis of selenoproteins including the transporter selenoprotein P (SELENOP) and extracellular glutathione peroxidase (GPX3). SELENOP deficiency in transgenic mice resulted in a Se status-dependent phenotype characterized by impaired growth and disturbed neuronal development, with epileptic seizures on a Se-deficient diet. Therefore, we hypothesized that Se and SELENOP deficiencies may be prevalent in paediatric patients with a neurodevelopmental disease. In an exploratory cross-sectional study, serum samples from children with neurodevelopmental diseases (*n* = 147) were analysed for total serum Se, copper (Cu), and zinc (Zn) concentrations as well as for the TE biomarkers SELENOP, ceruloplasmin (CP), and GPX3 activity. Children with epilepsy displayed elevated Cu and Zn concentrations but no dysregulation of serum Se status. Significantly reduced SELENOP concentrations were found in association with intellectual disability (mean ± SD (standard deviation); 3.9 ± 0.9 mg/L vs. 4.4 ± 1.2 mg/L, *p* = 0.015). A particularly low GPX3 activity (mean ± SD; 172.4 ± 36.5 vs. 192.6 ± 46.8 U/L, *p* = 0.012) was observed in phacomatoses. Autoantibodies to SELENOP, known to impair Se transport, were not detected in any of the children. In conclusion, there was no general association between Se deficiency and epilepsy in this observational analysis, which does not exclude its relevance to individual cases. Sufficiently high SELENOP concentrations seem to be of relevance to the support of normal mental development. Decreased GPX3 activity in phacomatoses may be relevant to the characteristic skin lesions and merits further analysis. Longitudinal studies are needed to determine whether the observed differences are relevant to disease progression and whether correcting a diagnosed TE deficiency may confer health benefits to affected children.

## 1. Introduction

Children are at risk for trace element (TE) deficits due to rapid growth and high metabolic demands [1,2,3,4]. A dysregulated TE metabolism can severely impair brain development and neurocognitive functions, eventually manifesting as a neurodevelopmental disease [5,6]. Among the different TE, selenium (Se) is of particular importance as it is essentially needed for the biosynthesis of several selenoproteins with key functions in metabolism, endocrine signalling, and antioxidative defence systems [7,8,9,10]. However, due to biogeochemical reasons, the concentrations and availability of Se in soil are unevenly distributed, and there is high risk for Se deficiency in many areas of the world [11,12]. An insufficient habitual intake of Se by nutrition is considered a relevant health issue in large areas of South America, Africa, Asia, Australia, and Europe, where a number of diseases have been associated with Se deficiency [13,14,15,16,17,18]. However, it appears impossible to predict Se intake by food patterns alone, and laboratory analyses are necessary for assessing Se status in populations and specific risk groups [19]. Importantly, Se deficiency constitutes an addressable deficit, and targeted supplementation with Se was successfully applied to reduce the incidence and prevalence of certain Se-dependent endemic diseases, e.g., Keshan and Kashin–Beck disease in central China [20,21]. By these nutritional measures, protection and health improvements were reported in both children and adults, and it is likely that the course of pregnancy, child birth, and early development are positively supported by supplemental Se in populations with Se deficiency [22,23,24]. Several reports from animal models suggest that selenoprotein expression is controlled by sexually dimorphic mechanisms, and that sensitivity to Se deficiency displays sex-specific characteristics [25,26,27,28]. The extent to which the same mechanisms also work in human subjects is not yet known.

Inherited defects in Se metabolism, selenocysteine (Sec) biosynthesis or selenoprotein expression have highlighted a high vulnerability of the brain to Se deficiency and an intricate dependence of neuronal survival on a sufficiently high Se supply, which was observed in both affected children and transgenic mouse models [7,8,9,10]. The first selenoprotein-related gene defect in human subjects was identified based on an atypical thyroid hormone pattern in blood, i.e., elevated thyroxine (T4) in the face of reduced tri-iodothyronine (T3), indicative of a conversion defect from impaired expression of the deiodinase family of selenoenzymes [29]. The affected gene *Secisbp2* turned out to encode selenocysteine insertion sequence (SECIS)-binding protein-2, i.e., an RNA-binding protein with an essential role for selenoprotein biosynthesis. Individuals with biallelic *Secisbp2* variants displayed a wide range of selenoprotein-related symptoms including growth defects, retarded bone development, metabolic dysregulation, enhanced ultraviolet (UV)-sensitivity, male infertility, muscular dystrophy, and sensorineural hearing loss [30,31,32]. Complete knockout of *Secisbp2* in mice had embryonic lethal consequences [33]. 

A similar dramatic phenotype has been described in children with biallelic variants of the phosphoseryl-tRNA:Sec-tRNA synthase (*SEPSECS*) gene, encoding the enzyme that catalyses the last step of Sec-tRNA biosynthesis. The affected children present with pontocerebellar hypoplasia type 2D, with reduced intellectual development, spasticity, ataxia, epilepsy, hypotonia, and progressive cerebello-cerebral atrophy [34,35,36]. Similarly, children with biallelic non-sense variants in the ubiquitously expressed Se-dependent phospholipid-hydroperoxide glutathione peroxidase (GPX4) displayed spondylometaphyseal dysplasia, Sedaghatian type [37]. The neurodevelopmental phenotypes are reminiscent of those observed in corresponding transgenic mouse models [38,39], or upon the genetic inactivation of the transporter selenoprotein P (SELENOP) under limited nutritional Se supply [40,41,42,43]. The defective maintenance of brain Se status, loss of privileged Se supply to the nervous system, or impaired expression of SELENOP may cause neurodegeneration, neurological symptoms, and developmental delay [7,8,9,43,44]. A few cases with systemic Se deficiency associated with neurodevelopmental diseases and epilepsy have been reported, and they responded positively to Se supplementation [45].

Given the current state of knowledge on TE and neurodevelopmental diseases, we postulated Se deficiency or low Se supply to brain by depressed circulating SELENOP levels to be prevalent in paediatric patients with neurodevelopmental diseases. Accordingly, we conducted a cross-sectional study with paediatric patients. Although the results do not support our major hypothesis, we identified novel and unexpected interrelations between the TE status and neurodevelopmental diseases that are of potential health relevance and in need of further clinical evaluation.

## 2. Materials and Methods

### 2.1. Study Design

A cross-sectional study was conducted at the Centre for Chronically Sick Children (SPZ) and the Department of Paediatric Neurology of Charité-Universitätsmedizin Berlin (Berlin, Germany) between 2017 and 2019. Paediatric patients were enrolled irrespective of diagnosis when a routine analytical blood drawing was scheduled. Leftover serum samples from paediatric patients (*n* = 147) were available for laboratory analysis and the assessment of TE status (Supplementary Materials Table S1). Clinical diagnosis was classified according to the International Classification of Diseases, tenth revision (ICD-10) (Table 1). Recruitment proceeded in consecutive manner and no specific inclusion or exclusion criteria were applied, in order to obtain an unbiased cross-sectional sample of the available paediatric patients. The study was approved by the ethical committee of Charité-Universitätsmedizin Berlin (#EA2/028/16), and at least one parent or legal guardian from all study subjects enrolled into the analysis provided a written informed consent form. The samples were deposited in a local biobank and stored at −80 °C until analysis. All measurements were conducted by scientists blinded to any clinical information.

### 2.2. Trace Element Analysis

Serum TE concentrations were quantified by total reflection X-ray fluorescence (TXRF) analysis using a benchtop TXRF analyser (S2 Picofox, Bruker Nano GmbH, Berlin, Germany), essentially as described previously [46]. Briefly, samples were diluted 1:2 (*v/v*) with a gallium standard (1000 µg/L), and 8 µL of the dilution was applied to the middle of a polished quartz glass slide and dried overnight. One serum standard served as control sample in each assay run for quality assessment. The inter- and intra-assay CV (coefficient of variation) were determined to be below 15% during the analyses.

### 2.3. Quantification of Protein Biomarkers of Se and Cu Status

Serum SELENOP concentrations were determined by sandwich ELISA using a validated commercial SELENOP-specific ELISA (selenOtest, selenOmed GmbH, Berlin, Germany) [47]. Briefly, serum samples were pre-diluted 1:33 (*v*/*v*) with sample buffer and applied to antibody-pre-coated 96-well plates. Measurements were performed according to the manufacturer’s instructions. Standards and controls were included into each assay run for calibration and quality control. The inter- and intra-assay CV were determined to be below 11% during the analyses. 

Serum ceruloplasmin (CP) concentrations were quantified by a validated non-competitive sandwich ELISA using newly generated monoclonal antibodies, as described recently [48]. Briefly, serum samples were pre-diluted 1:300 (*v*/*v*) in sample buffer, and duplicates of the dilutions were incubated in antibody pre-coated ELISA plates for 30 min at room temperature. After several wash steps, the plates were incubated with detection antibody conjugated with horseradish peroxidase for 30 min. Following further wash steps, the enzymatic detection was started by adding 100 µL of tetramethylbenzidine (TMB) substrate and terminated by adding an equal volume of sulfuric acid. Absorption at 450 nm as readout was recorded by a spectrophotometric microplate reader (Tecan Group AG, Männedorf, Switzerland). Mean coefficient of variation of the serum samples analysed was at 7.3 ± 4.8%. 

Serum GPX3 activity was analysed by a coupled enzymatic test procedure monitoring the reduced nicotinamide adenine dinucleotide phosphate (NADPH) consumption at 340 nm as readout [49]. Briefly, serum samples of 5 µL were applied to 96-well plates on ice. After adding 200 µL of a test mixture including 1 mM NaN_3_ (sodium azide), 3.4 mM reduced glutathione, 0.3 U/mL glutathione reductase, and 0.27 mg/mL NADPH, the plates were transferred to a microplate reader (Tecan Group AG, Männedorf, Switzerland) and pre-incubated until 25 °C was reached. Measurements at 340 nm were then started by adding 10 µL of diluted hydrogen peroxide (0.00375%) as substrate. The decrease in NADPH absorbance per minute measured at 340 nm was proportional to the GPX3 activity in the sample. A serum sample was included as standard into each assay run for quality control. The inter- and intra-assay CV were below 20% during the analyses. 

### 2.4. Statistical Analysis

Statistical analysis was performed with SPSS Statistics^®^ (version 25, IBM, Chicago, IL, USA) and GraphPad Prism (Version 7, GraphPad Software Inc., San Diego, CA, USA). Normal distribution of values was tested by the Shapiro–Wilk test. Comparisons between two gender groups were conducted by unpaired t test, and the Mann–Whitney U test was used for non-normally distributed variables. Comparisons of the characteristics between more than two groups were conducted with ANOVA and Dunn’s multiple comparisons test, and for non-normally distributed variables with the Kruskal–Wallis test. Univariate analyses with a general linear model adjusted for age were used for comparisons between the diseased children and their control groups. The controls were the children in the study who did not have the specific disease, as serum from healthy children was not available for analysis for ethical reasons. Correlations were tested by Pearson’s correlation analysis, or by Spearman’s correlation test when data were not normally distributed. All statistical tests were two-sided and *p*-values < 0.05 were considered statistically significant; * indicates *p* < 0.05, ** *p* < 0.01, *** *p* < 0.001, and **** *p* < 0.0001. As this is an exploratory post-hoc analysis, all *p*-values are to be interpreted descriptively, and no adjustment for multiple testing was adopted.

## 3. Results

### 3.1. Patient Characteristics

The group of neuropediatric patients analysed contained more boys (*n* = 92, 63%) than girls (*n* = 55, 37%). The girls were on average slightly older than the boys (mean age 8.8 vs. 8.2 years), with an age span of 0–18 years (Supplementary Materials Table S1). The majority of children (76%) were diagnosed with an intellectual disability and/or a behavioural disorder. Many of the paediatric patients were diagnosed with more than one disease phenotype but were classified according to the main diagnosis (Table 1). For ethical reasons, it was not possible to recruit a group of healthy children as control. The analyses were therefore conducted by comparing the results of specific subgroups to the other paediatric patients combined. This approach enabled the identification of disease-specific abnormalities of TE or protein biomarkers, thereby highlighting particular metabolic characteristics or TE deficits or requirements. It allowed for a reliable direct comparison, as the same analytical technology was used and the samples had been collected, stored, and processed with identical pre-analytical histories. 

### 3.2. Trace Element Status in Relation to Age and Sex

**Figure 1:**
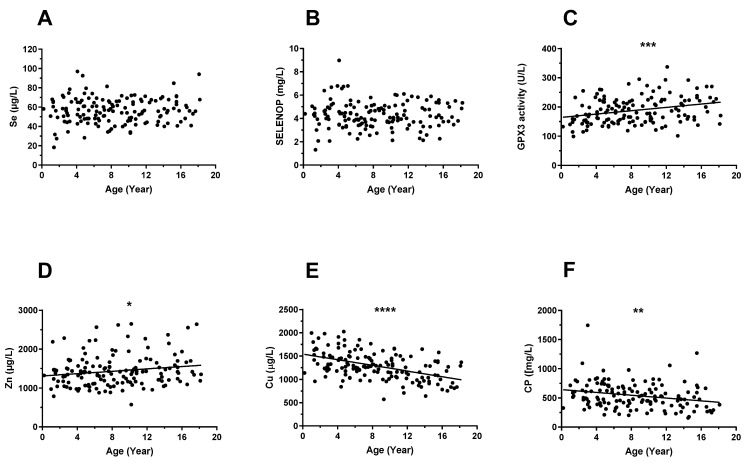
Correlations between age and biomarkers of trace elements in the paediatric patients. The analyses were comprising age and biomarkers of Se, Zn, and Cu status. There was no correlation between (**A**) age and serum Se, or (**B**) age and serum SELENOP concentration. Significant positive correlation was observed between (**C**) age and GPX3 activity (*r* = 0.292, *p* = 0.0004), and (**D**) age and serum Zn concentration (*r* = 0.146, *p* = 0.049). Strong negative correlation was observed between (**E**) age and serum Cu (*r* = −0.465, *p* < 0.0001) and between (**F**) age and CP concentration (*r* = −0.269, *p* = 0.002), indicating a declining Cu status with age. Correlation coefficients were calculated by Spearman test. Significant correlations are indicated by *p*-values; **** indicates *p* < 0.0001, *** indicates *p* < 0.001, ** indicates *p* < 0.01, and * indicates *p* < 0.05. SELENOP, selenoprotein P; GPX3, plasma glutathione peroxidase; CP, ceruloplasmin; Se, selenium; Zn, zinc; Cu, copper.

**Figure 2:**
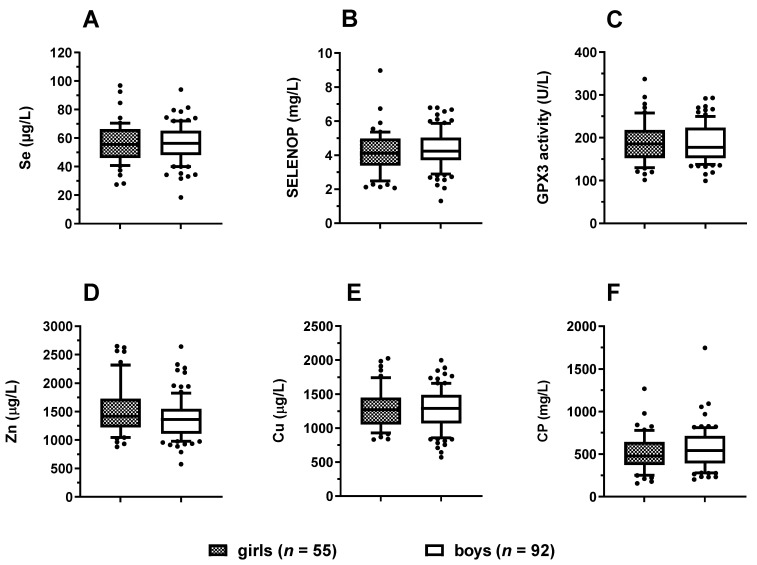
Comparison of serum TE (trace element) concentrations between female and male paediatric patients. All six TE biomarkers were compared between the groups of diseased girls (*n* = 55) and boys (*n* = 92). There were no significant sex-specific differences in (**A**) serum Se, (**B**) SELENOP concentrations, or (**C**) GPX3 activity. Similarly, (**D**) serum Zn, (**E**) Cu, or (**F**) CP concentrations did not differ between the sexes. Data are presented as box plots (10th–90th centiles); Student’s t-test or Mann–Whitney U test was used. All tests were two-sided and *p*-values < 0.05 were considered statistically significant.

To assess whether the TE status of the paediatric patients is age- or sex-related, the biomarkers were compared in relation to age (Figure 1) and in relation to sex (Figure 2). The analysis revealed no age-related differences in serum Se (Figure 1A) or SELENOP (Figure 1B) concentrations, whereas GPX3 activity correlated positively to age (Figure 1C). Total serum Zn concentrations showed a moderate positive correlation with age (Figure 1D). A relatively pronounced negative correlation was observed for both biomarkers of Cu status with age, i.e., serum Cu (Figure 1E) and serum CP (Figure 1F) concentrations.

Next, the patients were compared in relation to sex. No significant differences in serum Se (Figure 2A), SELENOP (Figure 2B), GPX3 activity (Figure 2C), Zn (Figure 2D), Cu (Figure 2E), or CP (Figure 2F) concentrations were observed between the girls and boys.

### 3.3. Correlations between Serum Cu, Zn, and Se Status and Protein Biomarkers

**Figure 3:**
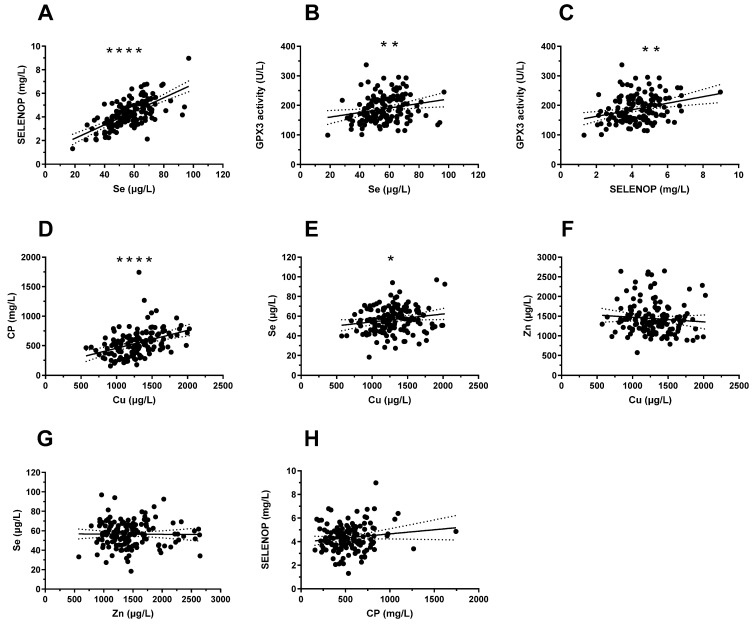
Interrelations between serum biomarkers of Cu, Se, and Zn status in paediatric patients. The analyses comprised three serum biomarkers of Se and two of Cu status, along with total serum Zn concentration. (**A**) The direct comparisons of Se and SELENOP (*r* = 0.655, *p* < 0.0001), or (**D**) Cu and CP (*r* = 0.389, *p* < 0.0001) yielded strong linear correlations over the full range of data. Similarly, (**B**) GPX3 activities and serum Se (*r* = 0.2507, *p* = 0.0026) as well as (**C**) GPX3 activities and SELENOP concentrations (*r* = 0.2495, *p* = 0.0027) showed positive correlations. A significant, albeit weak, interaction was observed between (**E**) Cu and Se. No interrelation was observed between (**F**) Cu and Zn, (**G**) Zn and Se, or (**H**) CP and SELENOP concentrations. Correlation coefficients were calculated by Pearson correlation test for normally distributed values, and by Spearman test for non-normally distributed values. Significance is indicated by *p*-values; **** *p* < 0.0001, ** *p* < 0.01, and * *p* < 0.05.

The serum Se status was evaluated by three complementary biomarkers, i.e., total serum Se and SELENOP concentrations, along with GPX3 activity. The Cu status was evaluated by two complementary biomarkers, i.e., total serum Cu and CP concentrations. Serum Zn status was assessed by total serum Zn concentrations. The assessment of the full cohort of samples showed a significant and linear correlation between serum Se and SELENOP over the full range (*r* = 0.655, *p* < 0.0001) (Figure 3A), as well as between total Cu and CP concentrations (*r* = 0.389, *p* < 0.0001) (Figure 3D). These results indicate that the serum samples were of high quality for laboratory analysis, and that the combination of total serum Se or Cu concentrations with the respective transport proteins SELENOP or CP yields congruent results in paediatric patients, as known from adult subjects. The children appeared to have a moderate Se status only, since the protein biomarker SELENOP was linearly associated with serum Se, and its expression was not saturated to maximal concentration levels. This notion was supported by the direct comparison of GPX3 activity with total serum Se (*r* = 0.2507, *p* = 0.0026) (Figure 3B) and GPX3 activity with SELENOP (*r* = 0.2495, *p* = 0.0027) (Figure 3C), yielding again linear correlations, albeit with less stringency than between Se and SELENOP. The correlation between serum Cu and CP was relatively strong, supporting the notion of a major contribution of CP to the systematic Cu status and Cu transport in children. In the group of paediatric patients, there was a significant, albeit weak, correlation between serum Se and Cu (Figure 3E) but no interrelation between SELENOP and CP (Figure 3H), Cu and Zn (Figure 3F), or Zn and Se (Figure 3G).

### 3.4. Trace Element Status in Relation to Epilepsy

**Figure 4:**
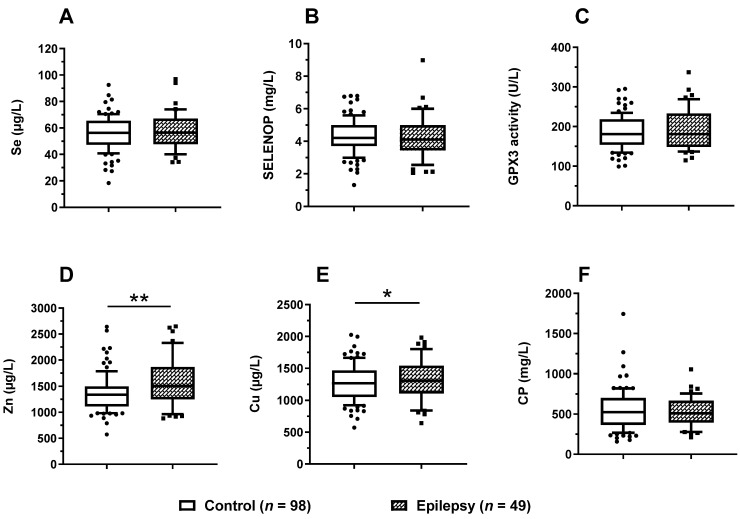
Comparison of serum biomarkers of TE status between paediatric patients in relation to epilepsy. All six TE biomarkers were compared between the groups of children with (*n* = 49) and without (*n* = 98) epilepsy. There were no significant differences in (**A**) serum Se, or (**B**) SELENOP concentrations, or (**C**) GPX3 activity between the two groups. (**D**) Serum Zn (*p* = 0.004) as well as (**E**) serum Cu levels (*p* = 0.023) were relatively elevated in the children with epilepsy, whereas (**F**) CP concentrations did not differ between the two groups. Data are presented as box plots (10th–90th centiles); general linear models with univariate test adjusted for age were used for analysis. *p*-values < 0.05 were considered statistically significant. ** indicates *p* < 0.01, and * indicates *p* < 0.05.

To test the major hypothesis, i.e., a potential relationship between a dysregulated Se status and the occurrence of epilepsy, all six TE biomarkers of Cu, Se, and Zn status were compared between patients with and without epilepsy (Table 1). Surprisingly, no significant differences between the groups were observed for total Se (Figure 4A), SELENOP (Figure 4B), or GPX3 activity (Figure 4C). However, children with epilepsy displayed significantly higher concentrations of serum Zn (Figure 4D) and slightly elevated Cu (Figure 4E) in comparison to the paediatric patients without epilepsy, whereas serum CP concentrations were not statistically different between the groups (Figure 4F).

### 3.5. Trace Element Status in Relation to Phacomatoses

**Figure 5:**
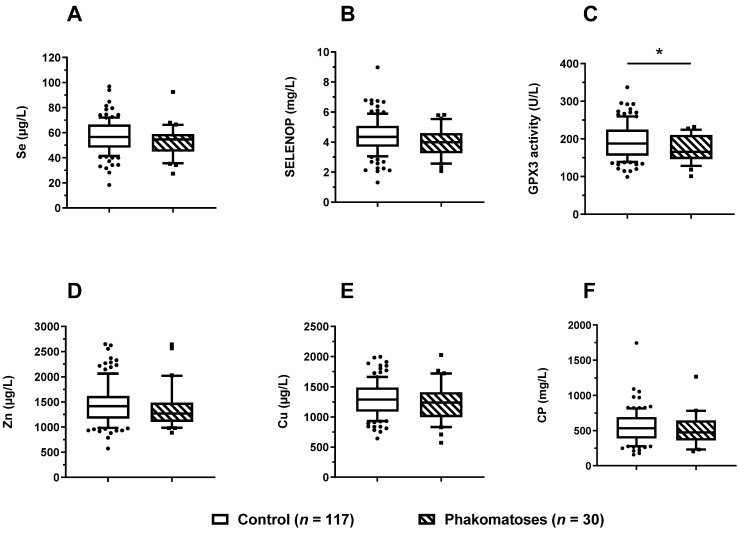
Serum TE status of paediatric patients in relation to phacomatoses. The cohort of paediatric patients was separated according to a diagnosis of phacomatoses. Patients with phacomatoses (*n* = 30) displayed a slightly lower but not significantly different (**A**) serum Se, and (**B**) serum SELENOP concentration, along with significantly lower (**C**) serum GPX3 activities (*p* = 0.012), as compared with the patients without phacomatoses (*n* = 117). No differences between the groups with or without phacomatoses were observed in relation to (**D**) serum Zn, (**E**) Cu, or (**F**) CP concentrations. Data are presented as box plots (10th–90th centiles); general linear model with univariate test adjusted for age was used. *p*-values < 0.05 were considered statistically significant; * indicates *p* < 0.05.

The cohort of patients encompassed a relatively high number of children with phacomatoses (*n* = 30). In direct comparison to the other patients, the children with phacomatosis displayed a tendency of Se deficiency, with a slightly lower serum Se and SELENOP status (Figure 5A,B). However, the differences (mean ± SD (standard deviation)) did not reach statistical significance; Se: 53.0 ± 12.4 vs. 57.4 ± 13.8 µg/L, *p* = 0.093, and SELENOP: 4.0 ± 1.0 vs. 4.4 ± 1.2 mg/L, *p* = 0.077, respectively. Serum GPX3 activity was significantly lower in the group of children with phacomatosis in comparison with the other paediatric patients (Figure 5C); GPX3: 172.4 ± 36.5 vs. 192.6 ± 46.8 U/L, *p* = 0.012). In comparison to the Se status biomarkers, there were no significant differences in serum Zn concentration (Figure 5D) or the biomarkers of Cu status (Figure 5E,F).

### 3.6. Trace Element Status in Relation to Mental Development

**Figure 6:**
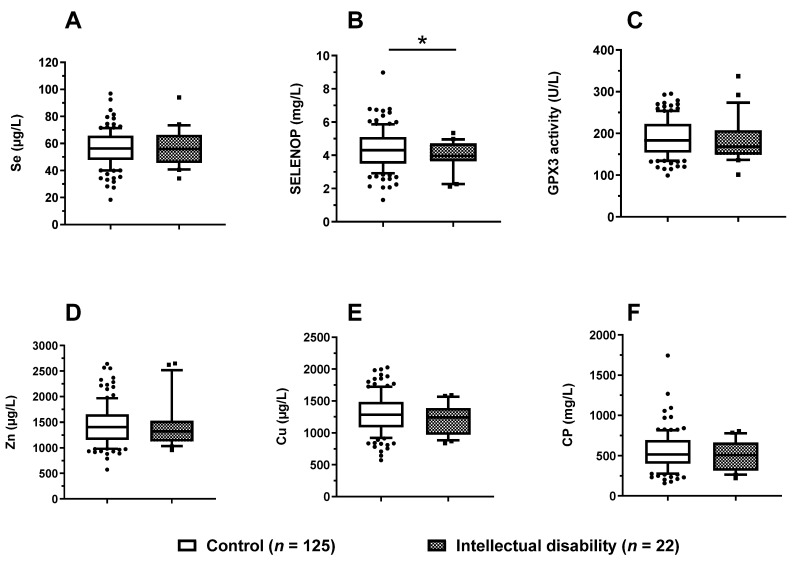
Serum TE status of paediatric patients with and without intellectual disability. The children with intellectual disability displayed no difference in (**A**) serum Se but (**B**) significantly lower serum SELENOP concentrations (*p* = 0.016) as compared with the children without intellectual disability. No differences in serum (**C**) GPX3 activities, (**D**) serum Zn, (**E**) serum Cu, or (**F**) serum CP concentrations were noted. Data are presented as box plots (10th–90th centiles); general linear model with univariate test adjusted for age and gender was used to compare the groups. All tests were two-sided and *p*-values < 0.05 were considered statistically significant; * indicates *p* < 0.05.

Finally, the paediatric patients were separated into a group of patients with an intellectual disability (*n* = 22) vs. the remaining paediatric patients (*n* = 125). Among all the six biomarkers of TE status analysed (Figure 6A–F), serum SELENOP showed a statistically significant difference (*p* = 0.016, and displayed a relative deficit in the children with an intellectual disability (Figure 6B). Notably, very few children displayed signs of very severe SELENOP deficiency, i.e., below the reference concentration threshold of 2.56 mg/L, as determined in adult subjects [50].

### 3.7. Trace Element Status of the Paediatric Patients in Relation to References

A comparison of the Cu, Se, and Zn concentrations in this cohort of neuropediatric patients with published reference values for a cross-sectional study on healthy children [51] or healthy European adults from a European cross-sectional analysis (EPIC) [46] indicates a relatively consistent and congruent result in relation to serum Cu and Se concentrations (Supplementary Materials Table S2). As compared to adults of the EPIC-study, the healthy children and the neuropediatric patients displayed on average a relatively lower Se status. The trend of decreasing serum Cu and CP concentrations with age in the cohort of paediatric patients as reported above (Figure 1E,F) was not observed in the cohort of healthy children, where total Cu as the sole Cu biomarker was analysed [51]. The Zn concentrations determined in the serum samples from the paediatric patients were consistently higher than in healthy adults and the corresponding age groups of healthy children. It is at present unknown whether this inconsistency relates to differences in the nutritional support of the patients, their origin of residency, or is due to technical or preanalytical issues.

## 4. Discussion

There is strong evidence from case reports and transgenic mouse models collectively indicating that impaired selenoprotein expression, low Se status and/or disturbed SELENOP biosynthesis, and the disrupted transport of Se by SELENOP to brain causes neurodevelopmental diseases associated with epileptic seizures, in some cases [10]. Consequently, we hypothesized that Se and SELENOP deficiencies are prevalent in children with impaired neurodevelopment, and that low Se status constitutes a particularly frequent finding in paediatric patients with epilepsy, especially in a European country with borderline Se supply like Germany. Our results do not fully support this hypothesis, and failed to indicate a particularly low general Se or SELENOP status in children with epilepsy. A strong Se deficit may still constitute an important, albeit rather rare reason for severe epilepsy in children. Nevertheless, the stringent linear correlations between serum Se concentrations with the protein biomarkers of Se status, i.e., serum SELENOP levels and GPX3 activities, respectively, argue for a marginal supply of the paediatric patients with the essential trace element Se, in good agreement with the data from adult European subjects who are also considered as insufficiently supplied [52]. However, a severe deficit, i.e., a Se status below the 2.5th percentile of a reference population of healthy European adults (serum Se < 45.7 µg/L or SELENOP < 2.56 mg/L, [53]), was only rarely observed in the paediatric patients analysed, in contrast to e.g., a study in Egypt, where strong Se deficiency in epileptic children was prevalent [54]. Moreover, autoantibodies to SELENOP, known to impair Se transport, were not detected in any of the children (not shown) [55]. An interesting and potentially relevant finding was noted in relation to general mental development, where disability was associated with a significantly depressed SELENOP status. This finding appears plausible in view that SELENOP constitutes the major transporter for Se into the central nervous system [42,43]. However, systematic clinical studies relating the Se status of neonates with their cognitive and motor development are missing, yet, some studies indicate a relevant role of a mother’s Se status for their child’s neurocognitive development [23,56] and pregnancy course [22,24]. Some more interesting findings were noted when testing additional hypotheses related to other TE and paediatric diseases, i.e., a relatively elevated Cu and Zn status in children with epilepsy, and a general trend towards low Se, SELENOP, and GPX3 levels in phacomatoses were noted. In particular, the latter finding may be of clinical relevance for the affected children in view of their generally increased tumour risk [57]. The analysis of serum iron (Fe) status in our cohort of children did not indicate differences in relation to disease, sex or age (Supplementary Materials Figure S1).

Phacomatoses are neurocutaneous syndromes mostly affecting the structures that arise from the embryonic ectoderm, i.e., the nervous system, skin, and eyes [58,59]. The interrelation with Se status may be two-fold, in view of the anti-oxidative and protective properties of selenoproteins. On the one hand, insufficient protection against oxidative stress and the reactive oxygen-mediated damage of DNA may contribute to phacomatosis development; having said that, progressive skin lesions are associated with increasing oxidative tone and pro-inflammatory cytokines collectively down regulating selenoprotein expression and status [48,60,61]. This suggests that a pre-existing Se deficiency with low expression of selenoproteins may contribute to the initiation of phacomatoses, and that a sustained depressed Se status may be of relevance to further disease development. Phacomatoses encompass a large group of different diseases including neurofibromatosis type 1 (peripheral NF), type 2 (central NF), tuberous sclerosis, and Von Hippel–Lindau syndrome [58,62], which is associated with cellular hypoxia [63]. Notably, besides pro-inflammatory cytokines and inflammation, hypoxia has been identified as another condition leading to reduced selenoprotein biosynthesis and Se status [64]. This interplay between Von Hippel–Lindau syndrome, hypoxia, and low expression of selenoproteins may close a self-amplifying cycle of progressive inflammation and damage, and may positively be affected by anti-inflammatory therapy in combination with adjuvant supplemental Se. Further studies are needed to clarify whether the incidence and symptom progression of phacomatoses is related to low Se, and whether the tumour risk can be positively modulated by avoiding severe Se deficiency.

Some prior observational studies have indicated that the serum concentration of some essential TE might be associated with age or gender [51,65,66]. However, no significant differences in Se, Zn, or Cu status were noted when comparing the male and female paediatric patients in our study. The Cu status decreased linearly with age in the group of children in this study, whereas it remained constant in healthy children of the same age ranges in a previous report [51]. The differences are not strong, and may be related to the origin of residency, or the clinical inflammatory state of patients. Unnoticed differences in Se metabolism and selenoprotein expression may be present in the children studied, especially in relation to age and sex, as shown in studies in rodent models [25,26,27,28]. However, the strongest differences were observed in the tissues and not necessarily in the blood, i.e., in a matrix that is not amenable to analysis in human subjects.

The particular strengths of the current study are the parallel assessment of different and coherent biomarkers of TE status by a standardized methodology, a relatively well-balanced cross-sectional collection of serum samples from all paediatric age groups, and the blinded set-up of the analyses. Among the limitations are the relatively limited information on the nutritional status and dietary patterns of the children, their particular acute disease and inflammatory state, the single time-point of analysis, and the lack of a control group of healthy age-matched children. Prospective studies linking disease course, severity, and acute episodes of tumorigenesis or seizures with the particular TE status are needed in order to better define the relevance of the TE supply and status to the different paediatric diseases of boys and girls and the disease progression, and importantly, for a better and laboratory-based nutritional counselling of the affected families.

## 5. Conclusions

We conclude from this study that a subset of paediatric patients displays a suboptimal status of essential TE, as reflected, for example, in the linear correlation of serum Se and SELENOP concentrations. However, and in contrast to expectations, the group of children with epilepsy was not particularly Se-deficient, thereby excluding Se deficiency as a prevalent consequence of epileptic seizures. The relatively low Se status observed in children with phacomatoses constitutes a novel and relevant finding, as both disease onset and propagation may be accelerated or aggravated by an insufficient expression of protective selenoenzymes. Further prospective studies are needed to better assess the potential relevance of the TE supply in paediatric patients, and to test whether the correction of a diagnosed deficiency in a single TE or a set of essential TE could be of value in positively influencing the course of the diseases and ameliorating disease episodes, symptoms, and progression. It is not unlikely that a severe deficiency in an essential TE constitutes a risk factor for a severe disease course, due to the high metabolic and anabolic demands of the young patients, especially in view that adjuvant supplementation with small dosages of sufficient amount to avoid TE deficiency is safe, well-tolerated, and inexpensive. The alternative of failing to respond to the TE demands of young patients due to incomplete analytical workup may profoundly hinder therapeutic success and positive neurological and motor development, as known from case reports, preclinical models, and clinical studies.

## Figures and Tables

**Table 1:** Main diagnosis of the Paediatric Patients (*n* = 147).

ICD-10 Code	Disease/Syndrome	Number of Children (%)
G40	Epilepsy	49 (33.3)
Q85	Phacomatoses	30 (20.4)
F70–F79	Intellectual disability	22 (15.0)
diverse	Diverse phenotypes	46 (31.3)

ICD-10, International Classification of Diseases, tenth revision.

## Data Availability

The data presented in this study are available on request from the corresponding author. The data are not publicly available due to data safety reasons.

## Supplementary Materials

The following supporting information can be downloaded at: https://www.mdpi.com/article/10.3390/nu14122375/s1, Figure S1: Analysis of serum Fe in pediatric patients with neurodevelopmental diseases; Table S1: Overview on sex and age of the pediatric patients and their ICD-10 diagnoses; Table S2: Comparison of serum TE levels of pediatric patients with published references.
