# Supplementary material for: Selenium Status in Paediatric Patients with Neurodevelopmental Diseases
Source: Nutrients. 2022 Jun 8;14(12):2375. doi: 10.3390/nu14122375 (PMC9227519; doi:10.3390/nu14122375)
Supplement: Supplementary file 1 [file nutrients-14-02375-s001.zip › nutrients-1747593-supplementary.pdf]

Table S1. Overview on sex and age of the pediatric patients and their ICD-10 diagnoses.

	Age (years, mean ± SD)	*n*
**males**	8.2 ± 4.7	92
**female**	8.8 ± 4.7	55
**sum**	8.4 ± 4.7	147
**ICD-10 code**	**Diseases**	***n***
**C00–D48**	Neoplasms	8
**D50–D53**	Nutritional anaemias	1
**E00–E90**	Endocrine, nutritional and metabolic diseases	25
**F00–F99**	Mental and behavioural disorders	112
**F70–F79**	Intellectual disability	22
**F80–89**	Disorders of psychological development	102
**G00–G99**	Diseases of the nervous system	70
**G40**	Epilepsy	49
**G80–G83**	Cerebral palsy and other paralytic syndromes	20
**H00–H59**	Diseases of the eye and adnexa	10
**H60–H95**	Diseases of the ear and mastoid process	9
**I00–I99**	Diseases of the circulatory system	17
**K00–K93**	Diseases of the digestive system	4
**M00–M99**	Diseases of the musculoskeletal system and connective tissue	11
**P00–P96**	Certain conditions originating in the perinatal period	20
**Q00–Q99**	Congenital malformations, deformations and chromosomal abnormalities	74
**Q00–Q07**	Congenital malformations of the nervous system	26
**Q20–Q28**	Congenital malformations of the circulatory system	7
**Q50–Q56**	Congenital malformations of genital organs	6
**Q65–Q79**	Congenital malformations and deformations of the musculoskeletal system	13
**Q85**	Phacomatoses, not elsewhere classified	30
**Q90–99**	Chromosomal abnormalities, not elsewhere classified	14
**R00–R99**	Symptoms, signs and abnormal clinical and laboratory findings, not elsewhere classified	12

ICD-10, International Statistical Classification of Diseases and Related Health Problems 10th Revision; SD, standard deviation.

Table S2. Comparison of serum TE levels of pediatric patients with published references.

	Age Group	*n*	ref. values, healthy children * Mean ± SD (µg/L)	*n*	neuropediatric values, this study Mean ± SD (µg/L)
**Cu**	**1 m–18 y**	**137**	**1296.3 ± 311.4**	**147**	**1288.1 ± 293.6**
	0–<1	18	876.9 ± 317.7	2	1568.0 ± 605.1
	1–<2	15	1340.8 ± 292.3	9	1539.8 ± 278.4
	2–<4	23	1366.2 ± 247.8	16	1395.6 ± 234.2
	4–<6	19	1232.8 ± 336.8	26	1413.6 ± 301.7
	6–<10	25	1487.8 ± 158.9	39	1294.1 ± 246.3
	10–<14	21	1340.8 ± 235.1	31	1187.7± 263.5
	14–<18	17	1302.7 ± 279.6	24	1082.4 ± 252.3
	**adults (EPIC) ****	**2069**	**1371.6 ± 319.1**		
**Zn**	**1 m–18 y**	**133**	**823.8 ± 232.1**	**147**	**1437.8 ± 406.3**
	0–<1	18	804.2 ± 255.0	2	1150.4 ± 248.2
	1–<2	15	758.4 ± 222.3	9	1356.5 ± 386.4
	2–<4	23	719.2 ± 215.8	16	1385.9 ± 350.5
	4–<6	19	836.9 ± 176.5	26	1364.1± 344.3
	6–<10	25	882.6 ± 202.7	39	1458.9 ± 447.2
	10–<14	21	869.6 ± 307.3	31	1411.3 ± 399.3
	14–<18	17	882.6 ± 209.2	24	1606.5 ± 443.4
	**adults (EPIC) ****	**2069**	**976.3 ± 293.7**		
**Se**	**1 m–18 y**	**130**	**60.8 ± 26.8**	**147**	56.5 ± 13.1
	0–<1	18	32.4 ± 18.2	2	54.4 ± 5.3
	1–<2	15	45.8 ± 15.0	9	49.2 ± 18.7
	2–<4	23	58.5 ± 24.5	16	57.9 ± 12.7
	4–<6	19	67.9 ± 30.8	26	59.9 ± 15.4
	6–<10	25	73.4 ± 24.5	39	54.7 ± 11.2
	10–<14	21	71.1 ± 23.7	31	55.5 ± 11.6
	14–<18	17	71.9 ± 25.3	24	58.8 ± 13.3
	**adults (EPIC) ****	**2069**	**84.7 ± 23.3**		

* Reference values for children as reported by Rukgauer et al. [51]; ** Reference data from a European cross-sectional analysis (EPIC) [46]. Ref., reference, TE, trace element; m, month; y, year; Cu, copper; Zn, zinc; Se, selenium.

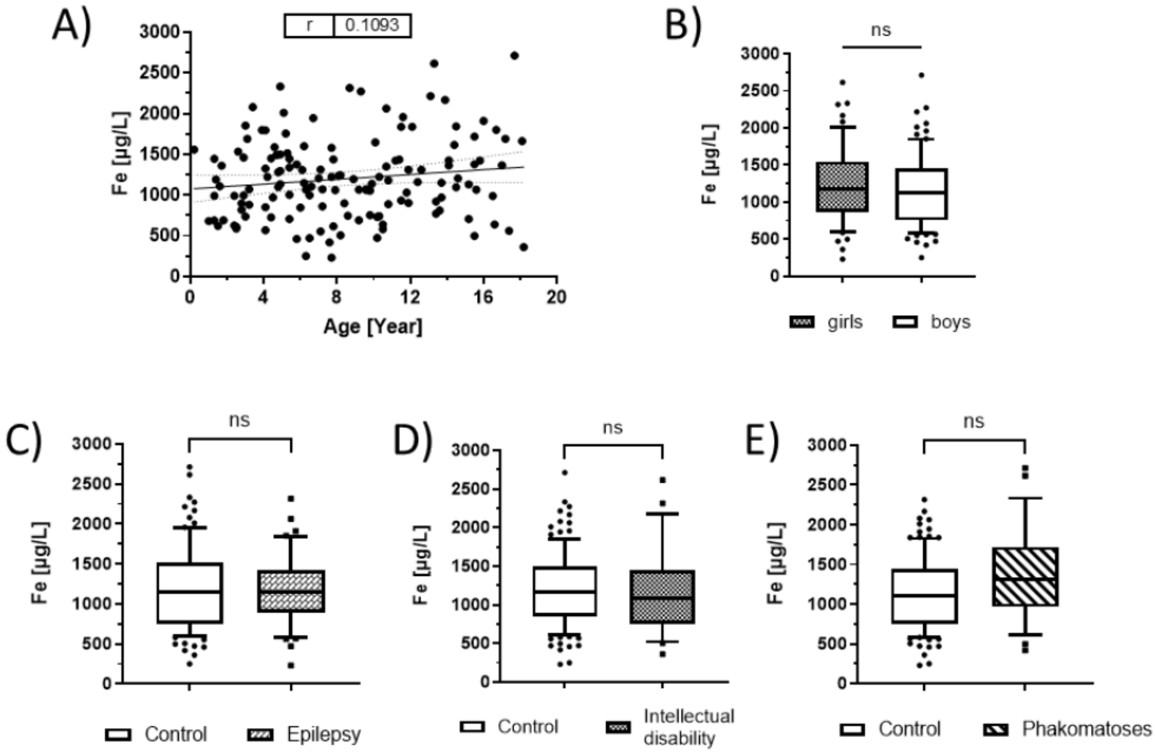

Figure S1. Analysis of serum Fe in pediatric patients with neurodevelopmental diseases. Serum iron (Fe) was not significantly associated with **(A)** age, **(B)** sex, **(C)** epilepsy, **(D)** intellectual disability, or **(E)** phakomatoses in the group of pediatric patients analysed in our study. ns, not significant.
